# Site-directed targeting of transcriptional activation-associated proteins to repressed chromatin restores CRISPR activity

**DOI:** 10.1063/1.5127302

**Published:** 2020-01-14

**Authors:** René Daer, Fatima Hamna, Cassandra M. Barrett, Karmella A. Haynes

**Affiliations:** 1 School of Biological and Health Systems Engineering, Arizona State University, Tempe, Arizona 85281, USA; 2 Eccles Institute of Human Genetics, University of Utah, Salt Lake City, Utah 84112, USA; 3 Wallace H. Coulter Department of Biomedical Engineering, Emory University, Atlanta, Georgia 30322, USA

## Abstract

Previously, we used an inducible, transgenic polycomb chromatin system to demonstrate that closed, transcriptionally silenced chromatin reduces Cas9 editing. Here, we investigated strategies to enhance Cas9 editing efficiency by artificially perturbing closed chromatin. We tested UNC1999, a small molecule inhibitor that blocks enhancer of zeste homolog 2, an enzyme that maintains closed polycomb chromatin. We also tested DNA-binding, transiently expressed activation-associated proteins (AAPs) that are known to support an open, transcriptionally active chromatin state. When cells that carried a polycomb-repressed transgene (luciferase) were treated with UNC1999 or the AAP fusion Gal4P65, we observed loss of histone 3 lysine 27 trimethylation (H3K27me3), a silencing-associated chromatin feature, at the transgene. Only Gal4P65 treatment showed full restoration of luciferase expression. Cas9 activity, determined by insertion deletion mutations, was restored in Gal4P65-expressing cells, while no CRISPR enhancement was observed with UNC1999 treatment. CRISPR activity was also restored by other Gal4-AAP fusions that did not activate luciferase expression. Our results demonstrate the use of DNA-binding, activator-associated fusion proteins as an effective method to enhance Cas9 editing within polycomb-repressed chromatin.

## INTRODUCTION

CRISPR/Cas9, a DNA-cutting system derived from bacteria, is a popular tool for precise genome engineering.^1,2^ However, recent work has shown that chromatin, the RNA-DNA-protein complex that controls chromosomal organization and gene expression in mammalian nuclei,^3–5^ blocks access of Cas9 to certain DNA target sites *in vitro*^6–8^ and in mammalian cells.^9,10^ While Cas9-mediated gene editing has been successful in specific applications, the complex structure of chromatin and its variation in different mammalian cell types and at different genes can still pose a barrier to reliable and consistent use.^8–11^ Recently, several groups have investigated the impact of chromatin states on the binding and cutting of DNA by Cas9 guide RNA complexes (Cas9/gRNA).^8–11^ A series of *in vitro* studies used reconstituted nucleosomes to demonstrate that Cas9/gRNA binding and cleaving are completely blocked at nucleosome-bound DNA.^6–8^ It is important to note here that the synthetic sequence associates more tightly with nucleosomes than natural sequences.^12,13^ To investigate Cas9 activity within natural chromatin, we and others have used HEK293 cell lines to show that complexes made up of constitutive heterochromatin protein 1 (HP1)^9^ or facultative polycomb repressive complex (PRC)^10^ suppress and in some cases completely block, Cas9 binding and DNA editing. Chromatin-mediated inhibition of Cas9 has also been demonstrated *in vivo* (zebrafish).^14^

**FIG. 1.**
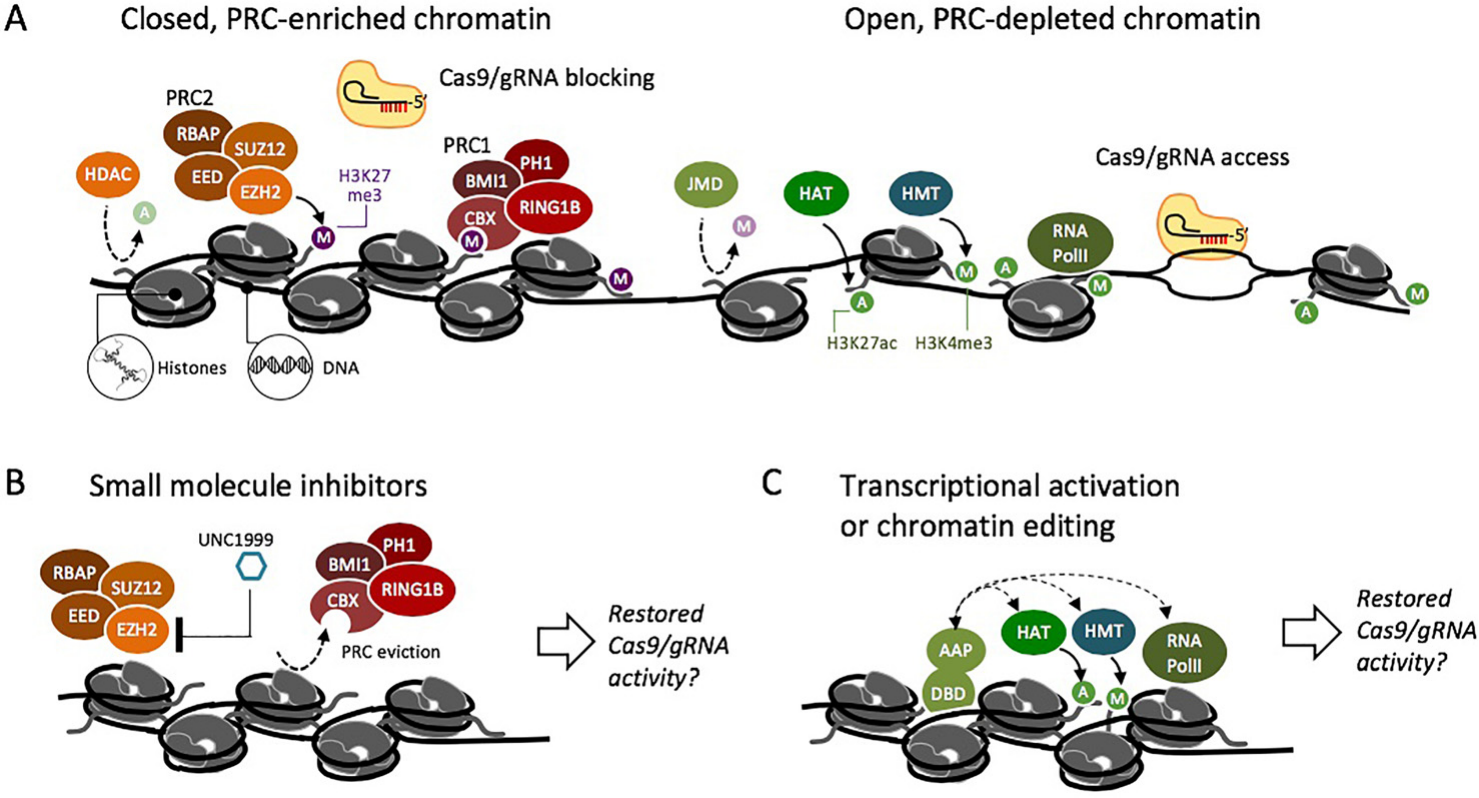
Research of Cas9 activity in chromatin suggests that facultative heterochromatin inhibits Cas9 editing, while open chromatin is permissive to Cas9. (a) PRC2 (polycomb repressive complex 2) generates the silencing mark histone 3 lysine 27 trimethylation (H3K27me3) (purple M). PRC2 includes suppressor of Zeste 12 (SUZ12), embryonic ectoderm development (EED), retinoblastoma-binding protein (RbAp), and enhancer of zeste 2 (EZH2).^3,5^ Polycomb repressive complex 1 (PRC1) includes chromobox protein homolog (CBX), ring finger protein 1b (RING1B), and polycomb group RING finger protein 4 (BMI1).^3,5^ Polycomb proteins support histone compaction and block access of DNA to RNA polymerase and Cas9.^9,10^ Chromatin remodelers, histone acetyltransferases (HATs), and histone methyltransferases (HMTs) generate modifications that support open chromatin, accessible DNA, and a transcriptionally permissive state.^23,24^ (b) Inhibitors of chromatin-modifying enzymes, such as UNC1999 that blocks EZH2, disrupt closed chromatin in a global, nonspecific manner.^25^ (c) Synthetic fusion proteins containing a DNA binding domain (DBD) and activation-associated protein (AAP) can be used to recruit open-chromatin-associated proteins to a specific locus.^26^

Scientists have explored the possibility of disrupting of closed chromatin to expose DNA to Cas9 and to enhance editing efficiency. Cas9 editing efficiency has been enhanced by proximal binding (within 100 bp) of nonenzymatic dCas9.^15,16^ Barkal *et al.* showed that the underlying mechanism is local chromatin remodeling.^16^ We have attempted to enhance CRISPR activity by artificially altering chromatin at a model transgene (“Tk-luciferase”) that had been packaged in PRC-enriched chromatin [Fig. 1(a)], which is found frequently throughout the genomes of multicellular organisms. Polycomb group (PcG) proteins and the silencing mark histone 3 lysine 27 trimethylation (H3K27me3) are critical for controlling gene expression during stem cell maintenance,^17,18^ differentiation, and oncogenesis.^19–22^ Polycomb-regulated genes are distributed over thousands of sites along chromosome arms in gene-rich regions. Therefore, PRCs may pose a substantial barrier to genome editing in humans and other animals. In our previous work, we demonstrated that siRNA-mediated knockdown of the PcG protein Suz12 was accompanied by an increase in Cas9-mediated editing efficiency at a luciferase transgene.^10^ Further investigation is needed to fully understand the conditions that are sufficient to generate a CRISPR-accessible state within closed chromatin. Therefore, we set out to explore additional methods to induce an open, Cas9-accessible state at sites within PRC-enriched chromatin.

Here, we broadened our investigation to two approaches for antagonizing closed chromatin at a PRC-enriched luciferase reporter: inhibition of the histone K27 methyltransferase enhancer of zeste homolog 2 (EZH2) [Fig. 1(b)] and DNA-binding fusion proteins that include a transcriptional activation associated protein (AAP) [Fig. 1(c)]. We measured changes in histone modifications and transcription levels following each treatment and then measured changes in Cas9 editing efficiency. We observed that targeting the transcriptional activator Gal4P65 to the silenced Tk-luciferase gene depleted the silencing-associated mark H3K27me3, increased Tk-luciferase expression, and increased Cas9 editing efficiency (insertion-deletions generated by nonhomologous end joining). We also found that other Gal4-AAP fusions that do not activate Tk-luciferase expression can increase Cas9 editing efficiency. Our results support the use of targeted fusion proteins as a general strategy for overcoming challenges associated with site-specific Cas9 inaccessibility.

## RESULTS AND DISCUSSION

### A chromatin enzyme inhibitor and a transcriptional regulator generate different states at a PRC-silenced reporter

**FIG. 2.**
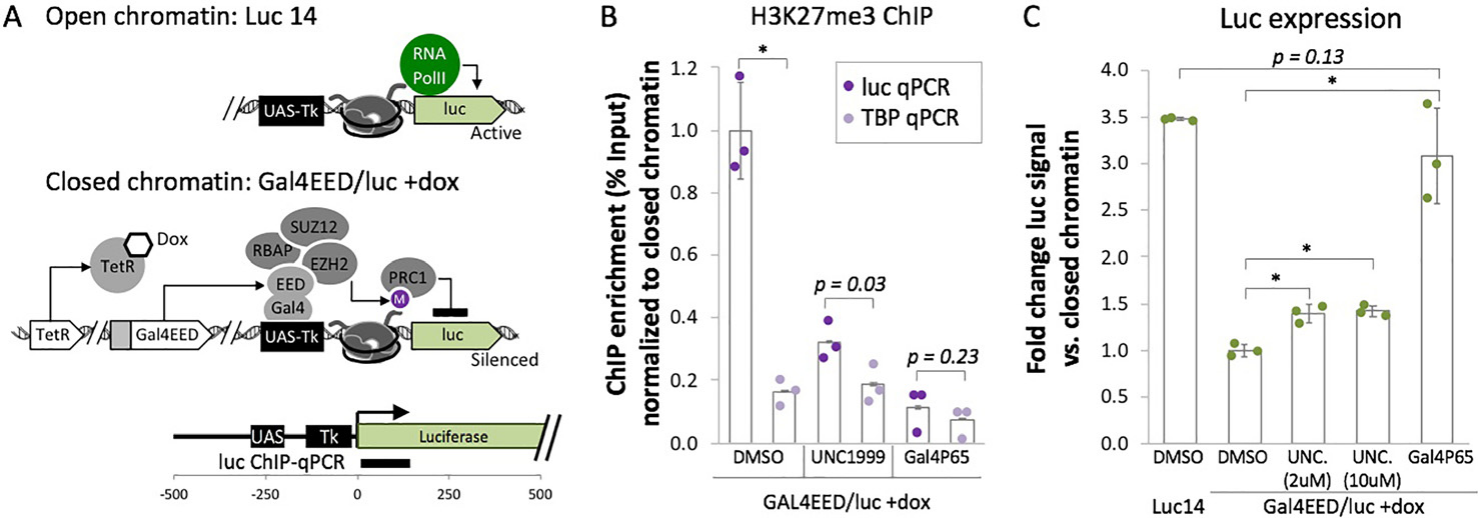
H3K27me3 and gene expression levels at the ectopic PRC-silenced Tk-luciferase reporter after treatment with EZH2 inhibitor UNC1999 or Gal4P65. (a) Open chromatin is characterized by expression of Tk-luciferase and absence of the silencing mark histone 3 lysine 27 trimethylation (H3K27me3).^10^ Closed chromatin is induced upon addition of doxycycline (dox) and is characterized by reduced expression of Tk-luciferase and accumulation of H3K27me3. (b) Chromatin immunoprecipitation followed by quantitative PCR (ChIP-qPCR) was used to determine H3K27me3 levels at Tk-luciferase after treatment with UNC1999 or expression of Gal4P65 compared to the inhibitor vehicle control DMSO. A constitutively active housekeeping gene, TATA-binding protein (TBP), was used as a negative control for H3K27me3 enrichment. Dots represent replicate IPs from a single chromatin prep, each normalized by the mean ChIP enrichment value for closed chromatin (Gal4EED/luc +dox DMSO luc qPCR). (c) Expression of Tk-luciferase was determined by luciferase activity assays. Dots, 3 independent treatments normalized by the average value for closed chromatin (Gal4EED/luc +dox DMSO). In (b) and (c): wide bars, mean values; error bars, standard deviation, ^*^p <* *0.01.

We hypothesized that antagonists of closed chromatin would restore a CRISPR-permissive state at target DNA [Figs. 1(b) and 1(c)]. To test this hypothesis, we used HEK293T Luc14 and HEK293T Gal4-embryonic ectoderm development (EED)/luc cells as a model system so that both open and closed chromatin could be investigated at a single, well-characterized chromosomal locus.^10,27^ Luc14 cells carry a chromosomal “UAS-Tk-luciferase” reporter in open, polycomb-depleted chromatin [Fig. 2(a)].^27^ We used a primer walking analysis to determine that the random chromosomal integration previously performed by Hansen *et al.*^27^ had generated a transgene-genome boundary at 2050–2457 bp upstream of the promoter region (Fig. S1). Gal4EED/luc cells,^27^ generated from Luc14, transiently express a Gal4-embryonic ectoderm development (EED) fusion protein when the cells are treated with a small molecule (doxycycline). Gal4EED binds to a Gal4 enhancer sequence (UAS) upstream of Tk-luciferase [Fig. 2(a)] and recruits polycomb group proteins, including enhancer of zeste homolog 2 (EZH2), which results in the accumulation of H3K27me3 and transcriptional silencing of Tk-luciferase.

PRC-silenced Tk-luciferase has been extensively characterized in previous studies,^10,27^ but the state of this locus after PRC is inhibited or antagonized has not yet been investigated. Therefore, we analyzed the central PRC-associated histone modification H3K27me3 and gene expression levels during EZH2 inhibition with UNC1999^25^ or targeted activation with a UAS-binding Gal4P65 fusion protein.^10^ We induced repressive chromatin at Tk-luciferase with dox-supplemented, complete growth medium for 4 days. We then either treated cells with a sublethal dose of UNC1999 (10 *μ*M) or transfected cells with a plasmid that expressed Gal4P65. We determined the sublethal dose of UNC1999 with a dose response assay (data not shown) using concentrations that effectively depleted H3K27me3 in HEK293 cells (1, 2, 5, 10, 20, and 50 *μ*M), as previously reported by Konze *et al.*^25^ We performed chromatin immunoprecipitation followed by quantitative polymerase chain reaction (PCR) (ChIP-qPCR) to determine H3K27me3 occupancy and measured expression of the Tk-luciferase transgene with a luciferase activity assay.

We observed a sharp decrease in H3K27me3 in UNC1999-treated and Gal4P65-expressing cells compared to the dimethyl sulfoxide (DMSO) control [Fig. 2(b)]. H3K27me3 enrichment was sixfold higher (p < 0.01) at PRC-repressed Tk-luciferase than at the active housekeeping gene TATA-binding protein (TBP). 10.0 *μ*M UNC1999 was nontoxic and effectively depleted H3K27me3 at Tk-luciferase (*p *=* *0.03) or expression of Gal4P65 (*p *=* *0.23). Since H3K27me3 is critical for PRC1 accumulation, depletion of H3K27me3 suggests disruption of PRC-enriched chromatin. However, the UNC1999-treated cells did not show fully restored Luciferase activity [Fig. 2(c)]. This result suggests that UNC1999 can effectively remove the silencing mark H3K27me3 from the silenced transgene but it does not enable full transcriptional activation under the conditions tested here. This outcome is consistent with previous work from Lee *et al.* who showed that removal of the H3K27me3 silencing mark was not sufficient to activate gene expression in embryonic stem cells.^28^ It is possible that the persistence of silencing-associated features other than H3K27me3, such as DNA methylation, histone deacetylation,^29^ or a high density of nucleosomes, might maintain the silenced state at Tk-luciferase.

In contrast, Gal4P65-expressing cells showed a roughly threefold increase in luciferase activity [Fig. 2(c)] compared to the DMSO-treated closed chromatin state (p < 0.01). Furthermore, Gal4P65-induced expression levels were similar to those observed for the open chromatin state in Luc14 cells (*p *=* *0.13). This outcome is consistent with our previously reported results for Gal4P65 targeted at Tk-luciferase.^10^ To determine whether Gal4P65-mediated activation results in the appearance of active chromatin modifications, we used ChIP-qPCR to measure H3K4me3 at Tk-luciferase. The active Tk-luciferase gene in Luc14 cells and the housekeeping gene “TBP” showed similar levels of H3K4me3 (*p *=* *0.24) (Fig. S2). Silenced Tk-luciferase in Gal4EED/luc +dox showed 1.8-fold lower H3K4me3 than the TBP control (*p *<* *0.01). After Gal4P65 expression, H3K4me3 levels approached those observed for TBP (*p *=* *0.12). These results suggest that the accumulation of activation-associated chromatin accompanies gene reactivation. Although both EZH2 inhibition and the Gal4P65 activator deplete the silencing mark H3K27me3 at Tk-luciferase, they generate different chromatin states. One is H3K27me3-depleted but only partially activated (1.39-fold expression vs the silenced control), and the other is fully transcriptionally active.

### CRISPR editing activity is restored by Gal4P65

**FIG. 3.**
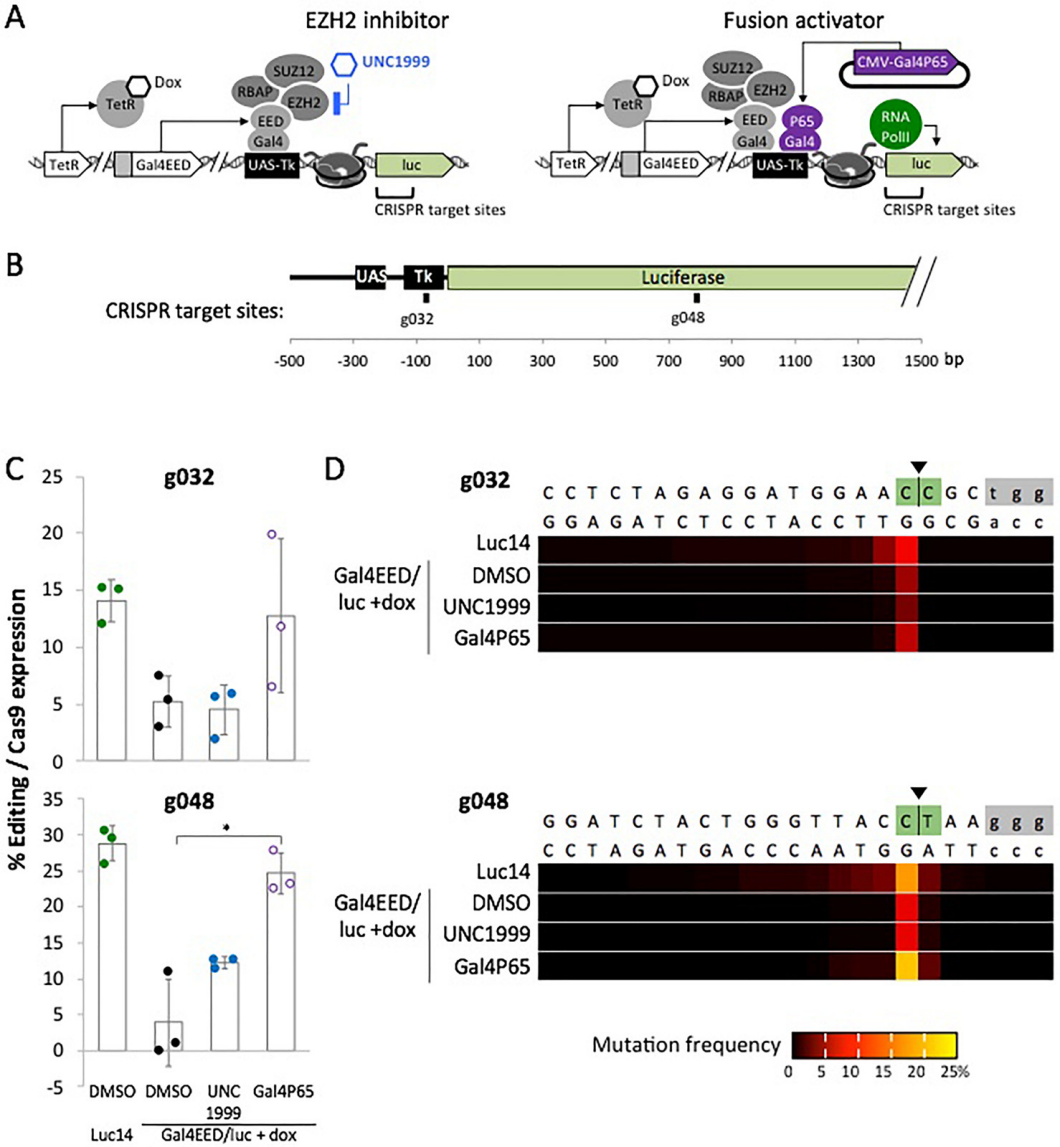
Effects of UNC1999 and Gal4P65 on Cas9-mediated editing in closed chromatin, determined by insertion-deletion mutations (INDELs) at Tk-luciferase. (a) Overview of the transgene states that were tested for CRISPR accessibility. Treatment of Gal4EED/luc +dox cells with UNC1999 inhibits enhancer of zeste 2 (EZH2), resulting in the loss of H3K27me3 [Fig. 2(b)]. Expression of Gal4P65 results in the loss of H3K27me3 and increased Tk-luciferase expression [Fig. 2(b)]. (b) Scaled map of the Tk-luciferase transgene and the gRNA target sites. (c) Charts show Cas9 editing efficiencies at each target site. Editing efficiencies were measured 3 days after transfection with Cas9/sgRNA plasmids. Dots, biological replicates; wide bars, mean values; error bars, standard deviation, ^*^p <* *0.01. (d) Heat maps indicate the frequency at which each DNA base position was affected by an insertion or deletion (INDEL).

Next, we investigated whether the observed changes in chromatin features and transcriptional states are accompanied by changes in CRISPR accessibility. We compared the generation of insertion or deletions (INDELs) via nonhomologous end joining repair (NHEJ) at Tk-luciferase in cells that were treated with the EHZ2 inhibitor UNC1999 or Gal4P65. Briefly, cells were treated with dox to induce closed chromatin and then treated with either vehicle control DMSO, UNC1999, or transfected with Gal4P65-expressing plasmid DNA [Fig. 3(a)]. Flow cytometry was used to determine Gal4P65 expression based on levels of the red fluorescent signal from the mCherry protein tag. One day later, we transfected the cells with plasmids that expressed enhanced green fluorescent protein (EGFP) and Cas9/sgRNA that targeted site g032 or g048 within Tk-luciferase [Fig. 3(b), for sequences see Methods]. These sites were chosen to represent regions where inhibition of Cas9 activity by closed chromatin was observed in our previous study.^10^ G032 is proximal to the closed chromatin nucleation site (within 300 bp of Gal4 UAS), while g048 is distal (roughly 1000 bp downstream). Three days after transfection, we collected the treated cells and assayed for Cas9 editing using deep sequencing to quantify the proportions of INDEL variants generated by NHEJ repair. We confirmed that mean editing efficiencies were reduced at g032 and g048 in closed chromatin of vehicle-treated (DMSO) cells compared to the open chromatin control (g032 and g048 *p *<* *0.01) [Fig. 3(c)].

In Gal4EED/luc +dox (closed chromatin) cells treated with UNC1999, Cas9 editing efficiency (INDEL formation) remained low at both target sites. The position of the most frequently edited sites (the nucleotide immediately adjacent to the predicted cut site) was also the same in UNC1999-treated cells and the controls [Fig. 3(c)]. In contrast, Gal4P65-expressing cells showed mean editing efficiencies that approached levels observed for open chromatin. The most significant enhancement of editing was observed at the distal site g048 (*p *<* *0.01 compared to Gal4EED/luc +dox DMSO). These results suggest that H3K27me3 depletion alone (induced by EZH2 inhibition) might not be sufficient to generate a CRISPR-accessible state at Tk-luciferase. In contrast, conversion to a gene expression-competent state, characterized as having H3K27me3-depleted and H3K4me3-enriched chromatin, as well as active transcription, supports Cas9-mediated editing.

### Activation-associated Gal4 fusions restore CRISPR-mediated editing

Activation of gene expression may not be desirable at CRISPR target genes that express RNA and proteins that are toxic to the cell or cause undesired changes in cell phenotype. This concern prompted us to investigate proteins with functions that are known to support an open chromatin state but are not sufficient to stimulate transcription. Transcriptional coactivators that interact with P65 include histone lysine acetyltransferases (HATs CREBBP and EP300), histone H3 lysine 4 methyltransferases (HMTs EHMT1 and SETD7), and others (STRING database^30^). To target these activities to silenced Tk-luciferase, we built Gal4 fusions that included previously characterized core domains ATF2 and KAT2B (HATs), KMT2A (HMT), as well as the chromatin remodeling complex subunit SMARCA4. The core activation-associated domain from each protein was cloned and expressed in frame with a Gal4 DNA binding domain (DBD) (Table S3). Expression of the fusion proteins in cells that carry the PRC-silenced Tk-luciferase gene (Gal4EED/luc +dox) had no impact on expression in luciferase activity assays.^32^

**FIG. 4.**
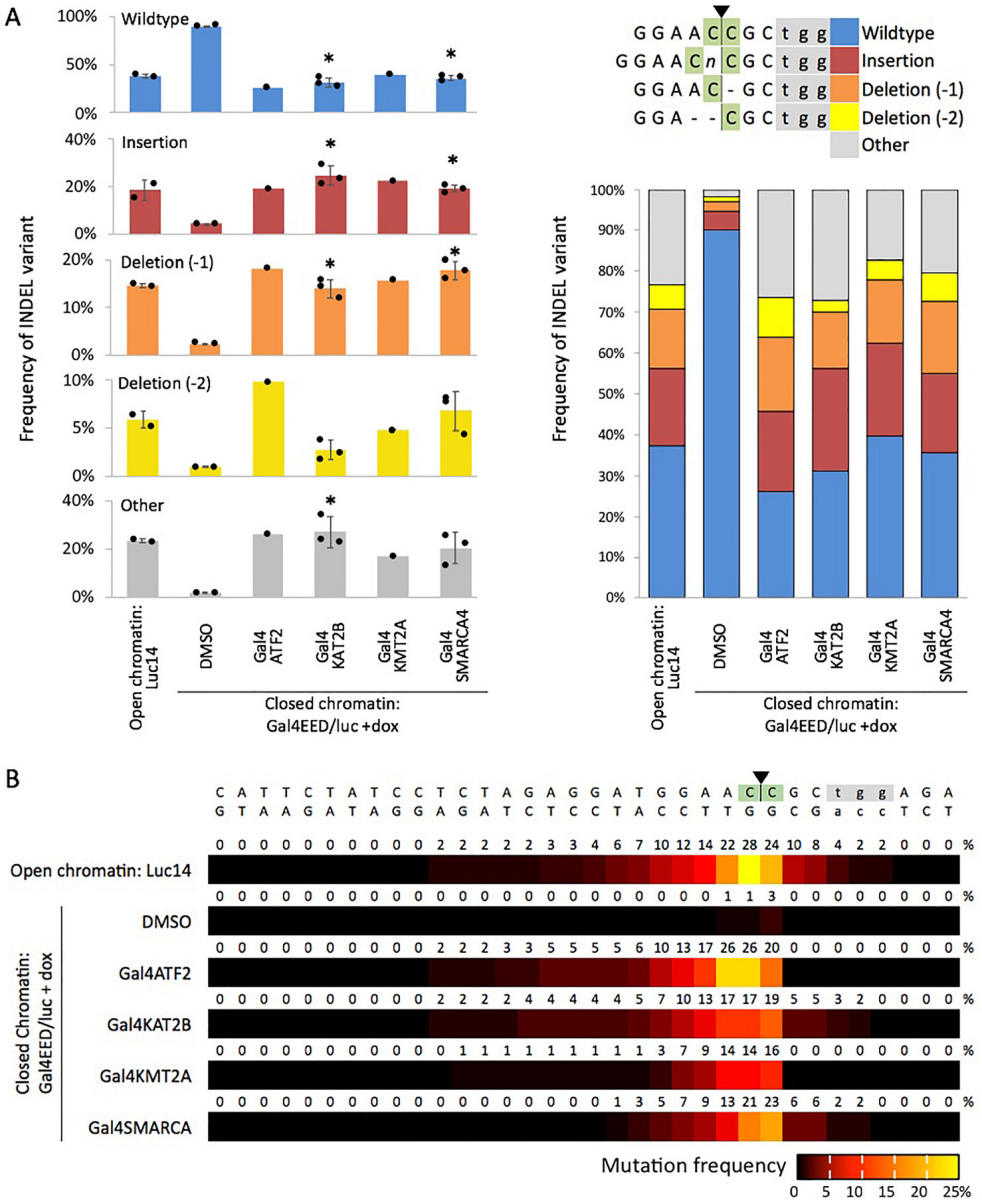
CRISPR activity is enhanced by Gal4 fusions that do not activate Tk-luciferase expression. (a) Frequencies of the four most common INDELs generated by NHEJ. In the bar charts, dots represent data from selected individual sequencing reactions where the knockout (KO) and Synthego ICE scores were greater than 40. Wide bars, mean values; error bars, standard deviation; ^*^p <* *0.01 for Gal4-fusions vs the closed chromatin control (Gal4EED/luc +dox DMSO). The stacked bar chart shows the distribution of INDEL variants (averaged values) for each sample. (b) Heat maps indicate the frequency at which each DNA base position was affected by an insertion or deletion (INDEL).

We targeted each Gal4 fusion to PRC-silenced Tk-luciferase (as described for experiments with Gal4P65) and determined editing efficiency (INDEL formation) at site g032. To enrich the samples for cells that expressed both a Gal4 fusion and Cas9, we used fluorescence activated cell sorting to collect mCherry- and EGFP-positive cells. We measured CRISPR-mediated editing (INDELs) after DNA extraction, PCR amplification, subsequent Sanger sequencing, and computational analysis with Synthego ICE software. For the open chromatin control (Luc14), the most prevalent INDELs included a one base pair mutation and one or two-base pair deletions next to the predicted cut site [Fig. 4(a)]. For the closed chromatin cells (Gal4EED/luc +dox +DMSO), we observed a much lower frequency of these INDELs (roughly 10%), and the majority of sequences were unedited.

In Gal4EED/luc +dox cells that expressed any of the four Gal4-AAP fusions, frequencies of single base pair insertions or single base pair deletions were restored to levels close to those observed for open chromatin [Fig. 4(a)]. Statistically significant increases (*p *<* *0.01) in these two types of INDELs were observed for the replicate sequencing data from Gal4KAT2B and Gal4SMARCA4-expressing cells compared to the closed chromatin control (Gal4EED/luc +dox +DMSO). The distributions of mutated base pairs in each Gal4-AAP-expressing sample also appeared to be consistent with the open chromatin control (Luc14) [Fig. 4(b)]. These results demonstrate that activation-associated proteins that do not activate expression of Tk-luciferase are sufficient to restore CRISPR editing to levels observed in open chromatin under the conditions reported here.

## CONCLUSIONS

To address barriers imposed by chromatin against efficient CRISPR-mediated editing of human DNA, we investigated methods for disrupting epigenetic silencing within polycomb protein-enriched chromatin. We compared two approaches: chemical inhibition of an enzyme that globally supports the formation of polycomb complexes and specific targeting of activation-associated proteins near the CRISPR target site. Since induced accumulation of PRC at Tk-luciferase blocks Cas9 binding and reduces editing via NHEJ,^10^ we hypothesized that removal of PRC and/or conversion to a transcriptionally active state would restore editing efficiency.

Inhibition of EZH2 via treatment with UNC1999 led to the depletion of H3K27me3 from the Tk-luciferase transgene (Fig. 2), but this was not sufficient to fully restore gene expression (Fig. 3) or enhance Cas9 editing. Our results support previous observations where removal of H3K27me3 did not result in complete transcriptional reactivation.^28^ Gene silencing and other repressive modifications can persist after the loss of PcG proteins.^29^ Therefore, the depletion of a silencing-associated mark may not be as effective as the direct modification of nucleosomes to improve DNA accessibility to Cas9-mediated editing. However, broad acting chromatin remodelers might be worth further investigation. They provide several potential advantages over fusion protein-dependent approaches. Inhibitors enable manipulation of chromatin at many sites at once, allowing for multiplex targeting of difficult sites. Low molecular weight compounds do not require transfection and can affect sites for which DNA-binding modules cannot be designed (e.g., low complexity sequences). Future work should investigate additional chromatin inhibiting drugs and drug combinations such as histone deacetylase (HDAC) inhibitors and DNA methyltransferase (DNMT) inhibitors for their impact on Cas9 activity in heterochromatin.

We were able to restore CRISPR-mediated editing by targeting a fusion transcriptional activator, Gal4P65, to PRC-silenced Tk-luciferase. In our previous study, we observed that CRISPR editing efficiency was reduced when Gal4P65 and dCas9/gRNA were coexpressed in a single transfection,^10^ suggesting steric hindrance of CRISPR by occupancy of the promoter region with activator proteins. For the new experiments reported here, we carried out transient expression of Gal4P65, and nine days later, we transfected the cells with dCas9/gRNA. Chromatin modifiers recruited by Gal4P65 or via transcriptional elongation might remove nucleosomes or silencing-associated marks over time to generate a CRISPR-accessible state. In our current report, we showed that targeted activation-associated peptides (AAPs) known to acetylate (ATF2, KAT2B), methylate (KMT2A), and remodel nucleosomes (SMARCA4) were sufficient to restore INDEL formation at the epigenetically silenced Tk-luciferase reporter. The AAP fusions appear to have a neutral effect on Tk-luciferase transcription,^31^ suggesting the potential to enhance access to CRISPR without stimulating unwanted changes in gene expression. Together, our results suggest that features such as acetylated histones, methylated histone H3 lysine 4, and/or nucleosome repositioning support access of Cas9 to DNA within a chromatin-silenced region. Our results also show that active transcription does not appear to be necessary to increase Cas9 editing efficiency. It is possible that physical occupancy by the fusion protein near the Cas9 target site is sufficient to increase access to the target DNA. Indeed, Chen *et al.* showed that target-proximal binding of dCas9 enhanced DNA editing by fnCas9.^15^ It will be important to profile chromatin features at CRISPR-enhanced sites in future work to ultimately define the molecular determinants of optimal CRISPR activity within chromatin. So far, we are the first to report that ATF2, KAT2B, KMT2A, and SMARCA4, which are associated with histone post-translational modification or remodeling, improve Cas9-mediated editing at closed, PRC-enriched chromatin.

An important advantage of targeted activation-associated fusions is the ability to affect a single genomic locus without disrupting other genes. To extend the utility of the fusions that we have tested at Tk-luciferase to endogenous sites, the Gal4 binding domain could be replaced with other customizable DNA binding modules such as zinc fingers (ZF), transcription activator-like (TAL) effectors, or dCas9/gRNA and be targeted to accessible DNA sites up- or down-stream of the desired editing target.^26^ The AAPs could also be tethered to Cas9 itself, similar to the approach recently reported by Ding *et al.* who enhanced Cas9 editing activity by fusing chromatin-modulating factors (high mobility group HMG and histone H1) directly to Cas9.^32^ Our characterization of histone modifier fusions further supports the burgeoning use of targeted, DNA-binding proteins to support Cas9-mediated editing in heterochromatin.

## METHODS

### Cell lines and plasmids

Cas9 transfections were done using pU6-(BbsI)_CBh-Cas9-T2A-EGFP (DNASU UnSC00746685) built from pX330-U6-Chimeric_BB-CBh-hSpCas9 (a gift from Feng Zhang, Addgene plasmid #42230) (described in Daer *et al.* 2017). The pU6-(BbsI)_CBh-Cas9-T2A-EGFP expresses the human optimized spCas9 protein and EGFP from the same mRNA transcript. Guide RNAs g032 and g048 were cloned into pU6-(BbsI)_CBh-Cas9-T2A-EGFP (described in Daer *et al.* 2017). Gal4 fusion transfections were performed with plasmid MV14, which was used to express each Gal4 fusion protein in-frame with an mCherry tag from a CMV promoter. Gal4 fusion-expressing plasmids were built by inserting PCR-amplified, XbaI NotI double digested core functional domains from p65, ATF2, KAT2B, KMT2A, or SMARCA4 into XbaI NotI digested MV14. See supplementary Table S4 for protein sequence references and primers with XbaI NotI extensions. The following Gal4 fusion plasmids are available at DNASU: Gal4P65_MV14 (HsCD00812387), Gal4ATF2_MV14 (HsCD00833013), Gal4KAT2B_MV14 (HsCD00833015), Gal4KMT2A_MV14 (HsCD00833016), and Gal4SMARCA4_MV14 (HsCD00833022). Sequences for each plasmid used in this study are available online with annotations at https://benchling.com/hayneslab/f_/V1mVw1Lp-chromatin-crispr-interference/.

### Cell culturing and transfection

Cell culturing, luciferase silencing, and transfections of the Luc14 and Gal4EED/luc HEK293 cell lines were performed as described in Daer *et al.* 2017. Briefly, cells were transfected in 12-well plates with Lipid-DNA complexes [500 ng plasmid, 3 *μ*l Lipofectamine LTX, 1 *μ*l Plus Reagent (Life Technologies)]. EZH2 inhibition was carried out with UNC1999 (Cayman Chemical) dissolved in DMSO to 10 mM. Gal4EED/luc cells were treated for four days with 1 *μ*g/*μ*l doxycycline to induce silencing. Cells were grown in dox-free media for one day and then treated with 10 *μ*M UNC1999 by diluting 1 *μ*l of 10 mM UNC1999 per 1 ml media for three days. Control cells were treated with 1 *μ*l of DMSO (vehicle) per 1 ml media. Cells were grown for one day in media without UNC1999 prior to luciferase assays, transfection with Cas9/sgRNA-expressing plasmids (described above), or ChIP-qPCR.

### Luciferase assays

Luciferase assay was performed in triplicate with a Synergy H1 Multimode Reader (Biotek) using the Steady-Luc Firefly HTS Assay Kit (Biotium) (described previously in Daer *et al.*).^10^

### Cas9 activity assays

Cells were collected after three days of growth following transfection with pU6-(BbsI)_CBh-Cas9-T2A-EGFP (DNASU UnSC00746685).^10^ Cas9 expression was determined by flow cytometry as described above and calculated as mean EGFP signal per cell. Genomic DNA was extracted from the remaining 200 *μ*l of cells using the QIAamp DNA Mini Kit (Qiagen) and eluted in 100 *μ*l of nuclease-free water. PCR of gDNA was performed using GoTaq 2× Mastermix (Promega) using primers P198/198 (see Table S3 for sequences) [95 °C for 10 min; 34× (95 °C, 30 s; 58 °C, 30 s; 72 °C, 20 s)]. Nested PCR was performed by diluting 2 *μ*l of PCR product into 500 *μ*l of water and Phusion High-Fidelity DNA Polymerase (Thermo-Scientific) (primers described in Tables S2 and S3). PCR reactions were purified using the Genelute PCR Cleanup Kit (Sigma-Aldrich). For the data shown in Fig. 3, DNA was submitted to the Center for Computational and Integrative Biology (CCIB) Core Facility (Massachusetts General Hospital) according to their requirements for sequencing. For the data shown in Fig. 4, the DNA was submitted to DNASU for Sanger sequencing. Three replicate sequencing reactions were performed per cell sample. The sequencing results were analyzed with Synthego®, ICE® Analysis v2.0 (https://ice.synthego.com) online using the gRNA target sequence and the following ab1 files as input: an 809 bp reference (control) sequence from unedited Luc14 and Sanger sequencing output (Cas9 edited sequences). Sequence data with knockout (KO) and ICE scores of greater than 40 and INDELs that occurred at 2% frequency or higher were included in the analysis. The sequence variants represented in the stacked bar chart (Fig. 4) were based on the most commonly observed INDELs across all samples. To generate the heat map shown in Fig. 4(b), mutant sequences were manually aligned with the unedited reference sequence. The frequencies displayed above each position were calculated as proportion of sequences that carried a change at each position. Data from replicate sequencing reactions were selected to calculate average frequencies.

### Quantitative PCR of ChIP DNA (ChIP-qPCR)

Chromatin Immunoprecipitation is described in the supplementary material. Relative quantification of ChIP samples was performed using real-time quantitative PCR and SYBR Green I master mix (Roche) as previously described.^10^ To adjust for input dilution, log2(20) was subtracted from input Cp values. Enrichment of H3K27me3 and H3K4me3 (%IP DNA bound of input) was calculated as 100 × 2̂(Cp_input_ − Cp_IP_).

Luciferase primer sequences:

P360 5′-CGGCGCCATTCTATCCTCTA-3′ (forward);

P361 5′-ATTCCGCGTACGTGATGTTC-3′ (reverse).

TBP primer sequences:

P363 5′-CAGGGGTTCAGTGAGGTCG-3′ (forward);

P364 5′-CCCTGGGTCACTGCAAAGAT-3′ (reverse).

### Statistical analyses

For the data in Fig. 3, the percentage of edited reads was normalized to the transfection efficiency for each sample. Averages and standard deviations were calculated for each of the *n *=* *3 biological replicates. The reported *p*-values were calculated using the two sample, one-tailed Student's t test in Microsoft Excel 16.16.10 using the formula T.TEST(array1,array2,1,2). For *p* < 0.01, 99% confidence with 2 degrees of freedom and a test statistic of t_(0.01,2)_ = 6.965 were determined.

Ethics approval was not required to perform this research.

R. Daer, K. Haynes, and Arizona State University Office of Technology Transfer have filed a provisional patent application for methods included in this manuscript.

## SUPPLEMENTARY MATERIAL

See the supplementary material for PCR analysis of the “pUAS-TK-luciferase” transgene (Fig. S1); H3K4me3 levels at the ectopic PRC-silenced reporter Tk-luciferase after treatment with EZH2 inhibitor UNC1999 or Gal4P65 (Fig. S2); UNC1999 kill curve (Table S1); list of primers for PCR of genomic DNA from Cas9/sgRNA treated samples (Table S2); sequences of primers from Table S2 (Table S3); and Method: crosslinked chromatin immunoprecipitation.

## NOMENCLATURE

PRC: Polycomb repressive complex
PcG: Polycomb group
